# Hallux valgus and hallux rigidus: a comparison of impact on health-related quality of life in patients presenting to foot surgeons in Australia

**DOI:** 10.1186/1757-1146-1-14

**Published:** 2008-12-11

**Authors:** Mark F Gilheany, Karl B Landorf, Priscilla Robinson

**Affiliations:** 1 Suite 4, 2nd Floor, Lansdowne House, 182–184 Victoria Parade, East Melbourne, Victoria 3004, Australia; 2 Podiatry Department, Faculty of Health Sciences, La Trobe University, Victoria 3086, Australia; 3 Musculoskeletal Research Centre, Faculty of Health Sciences, La Trobe University, Victoria 3086, Australia; 4 School of Public Health, Faculty of Health Sciences, La Trobe University, Victoria 3086, Australia

## Abstract

**Background:**

Hallux valgus and hallux rigidus are common foot conditions that lead to a deterioration in health status. Patients with significant pain or deformity from these conditions frequently resort to surgery. In this project, the foot health status of patients with hallux valgus and hallux rigidus presenting to foot surgeons in Australia was compared.

**Methods:**

Foot health status was measured in 120 participants using the Foot Health Status Questionnaire (FHSQ), a validated 0 – 100 point health status instrument. All participants had presented for surgical advice regarding hallux valgus/rigidus. The mean age of participants was 48.0 years (SD ± 14.3, range 19 – 79).

**Results:**

In the sample, 68% of participants were diagnosed with hallux valgus and 32% with hallux rigidus. Participants with hallux rigidus had greater levels of pain and functional limitation compared with hallux valgus. The mean difference for pain was 13.8 points (95% CI 4.6 to 22.9) and the mean difference for function was 15.0 points (95% CI 5.3 to 24.7). Both conditions result in similarly negative levels of impact on shoe fit and overall foot health.

**Conclusion:**

This study found measurable differences in foot health status between hallux valgus and hallux rigidus in participants presenting for surgical consultation. While both appear to have a negative impact on health status, hallux rigidus has a more significant impact.

## Introduction

Hallux valgus and hallux rigidus are two common pathologies that affect the first metatarsophalangeal joint. Both conditions can impact the joint to such an extent that reconstructive surgery may be required. Surgery to the 1^st ^metatarsal phalangeal joint is likely to be the most common joint surgery performed on the foot [1-4] and is the 4^th ^most common joint to be operated on behind the knee, hip and low back [2]. While affecting the same joint, the clinical and pathological profiles of hallux valgus and hallux rigidus are quite dissimilar.

Hallux valgus is a deformity of the first metatarsophalangeal joint. It is characterised by lateral drift of the great toe in association with joint subluxation. Occurrence rates for the condition reported in the literature vary, depending on the age of the participants investigated. Mann and Coughlin reported the frequency of hallux valgus in the adult shoe wearing population as 33% [5]. Similarly, Dawson et al observed a rate of 38% in women between 50 and 70 years [6]. In contrast, Menz and Lord report that in a sample of 71 older adults (aged 75 to 93) 70% were found to have hallux valgus [7].

Hallux rigidus is a deformity where there is a limitation to normal movements of flexion and extension (sagittal plane) leading to joint degeneration. It is recognised as a common form of osteoarthrosis in the foot [8] and has been described as affecting 10% of people aged 20–34 years and 44% of people over the age of 80 years [9]. The various aetiological factors in the development of hallux rigidus have been well described by Camasta [10], with trauma suggested as the most common cause of hallux rigidus. Many operative procedures and variations of procedures have been described for both hallux rigidus and hallux valgus [11].

Patient reported outcome measures are increasingly used to measure the impact of clinical conditions like hallux valgus and hallux rigidus on health status (health-related quality of life). Recently, one study examined the effect of hallux valgus on general health. Using the Medical Outcomes Study Short Form 36 (SF-36), Lazarides and colleagues found that hallux valgus results in a significant negative impact on general health [12]. Operative treatment of hallux valgus produces beneficial effects on general health-related quality of life [2,13-16].

Although some evaluation of the effects of hallux valgus on health status or health-related quality of life has been undertaken, the authors are not aware of any assessment of the effects on health status of hallux rigidus. Furthermore, the authors are unaware of any *comparison *of the impact of the two conditions (hallux valgus *versus *hallux rigidus) on foot health status. The aim of this study was, therefore, to describe and compare the foot health status of patients presenting to foot surgeons with hallux valgus and hallux rigidus.

## Methods

Between August and November 2002, 122 consecutive patients with a primary complaint of pain or deformity of the great toe joint were recruited from the offices of seven foot surgeons in Australia. Surgeons were recruited from the Australasian College of Podiatric Surgeons. Each surgeon received a detailed set of instructions in respect of patient selection and assessment (clinical and radiological). An evaluation sheet was also provided for recording diagnosis. Individuals were included in the study if they sought a surgical opinion with one of the aforementioned surgeons regarding great toe joint pain or deformity. Exclusion criteria included:

(i) Less than 18 years of age

(ii) Not fit for general anaesthesia

(iii) Unable to communicate fluently in English

(iv) Previous foot surgery.

Once enrolled in the study, participants' foot health status was evaluated to determine the impact of their hallux valgus or hallux rigidus deformity. Ethical approval was granted prior to the commencement of the study by the Faculty Human Ethics Committee, La Trobe University, Melbourne, Australia (FHEC01/207). All participants signed informed consent prior to recruitment.

### Outcome measure

Health status was measured using the Foot Health Status Questionnaire (a self-rated health status measure). It has been previously validated (content, criterion and construct validity) across a wide spectrum of pathologies including skin, nail and musculoskeletal disorders. It has a high test-retest reliability (intraclass correlation coefficients ranging from 0.74 to 0.92) and a high degree of internal consistency (Cronbach's α ranging from 0.85 to 0.88) [17]. The Foot Health Status Questionnaire has four domains covering foot pain, foot function, shoe fit (footwear) and general foot health. Each domain is rated on a scale of zero to one hundred, with a higher score indicating better foot health (i.e. 0 = worst foot health and 100 = best). Participants were asked to complete the Foot Health Status Questionnaire at the time of recruitment.

### Clinical diagnosis

Diagnosis was delineated into 'hallux valgus', 'hallux rigidus' or 'other' based on clinical and radiographic evaluation. Hallux valgus was recorded as Stage 1 – 4 based on the criteria as described by Root et al 1977 [18] (Table 1). Hallux rigidus was recorded as Grade 1 – 3 using the classification for degeneration in the 1^st ^metatarsophalangeal joint as described by Regnauld [19] (Table 2).

**Table 1:** Stages of Hallux Valgus as defined by Root et al 1977

**Stage 1**
Lateral displacement of the great toe (hallux) at the metatarsophalangeal joint
Minimal sesamoid displacement
**Stage 2**
Hallux abductus deformity (great toe pressing against the 2^nd ^toe)
Sesamoid displacement apparent

**Stage 3**
Increased intermetatarsal angle
Sesamoid displacement to level of partial disarticulation with the metatarsal head
Possible associated 2^nd ^hammertoe and bony adaptation of metatarsal head

**Stage 4**
Partial/complete dislocation of hallux at metatarsal phalangeal joint.
Hallux under-riding or over-riding lesser toes

**Table 2:** Grade of 1^st ^metatarsophalangeal joint degeneration as defined by Regnauld 1986

**Grade 1**
Functional limitation of dorsiflexion, mild dorsal spurring, acute/subacute pain joint enlargement, slight joint space narrowing, no structural sesamoid disease
**Grade 2**
Limitation of motion (75%), broadening and flattening of the metatarsal head and base of proximal phalanx, joint space narrowing, structural first ray elevatus, osteochondral defect, metatarsalgia, sesamoid hypertrophy

**Grade 3**
Severe loss of joint space, ankylosis, extensive dorsal medial and lateral spurring, osteophytes may bridge the joint space, osteochondral defects of metatarsal head +/- proximal phalanx +/- joint mice, extensive sesamoid hypertrophy

### Sample size, data handling and analysis

Although 122 participants were initially recruited, data from one individual were incomplete and one participant was eventually diagnosed with sesamoid pathology. Both participants' data were removed from the final analysis, leaving a total of 120.

Comparison of Foot Health Status Questionnaire scores between the hallux valgus and hallux rigidus groups were performed using independent sample t-tests. Analysis was performed using SPSS Version 11 (SPSS Inc, Chicago, IL) and statistical significance was set at p < 0.05.

## Results

The majority of participants (82%) were female. The mean age was 48.0 years (SD ± 14.3, range 19 – 79) and 60% of participants were between the ages of 45 and 64 years. There was no significant difference in the age of participants in both groups (47.4 years ± 15.5 for hallux valgus versus 49.9 years ± 10.7 for hallux rigidus, p = 0.36). Most participants (57%) had no co-morbidities, such as hypertension and asthma; with 22% reporting 1 co-morbidity, 10% reporting 2 co-morbidities, and 11% reporting between 3 and 6 co-morbidities.

In the sample, 82 out of 120 (68%) were diagnosed with hallux valgus and the remaining 38 (32%) were diagnosed with hallux rigidus. Females were more likely to present with hallux valgus, while males were more likely to present with hallux rigidus. Of the females in the study (n = 99), 76% had hallux valgus and 24% had hallux rigidus. In contrast, of the males in the study (n = 21), only 33% had hallux valgus while 67% had hallux rigidus.

The median stage of deformity (as defined by Root [18]) for participants presenting with hallux valgus was 3 (range 1 – 4). The median grade of deformity (as defined by Regnauld [19]) for participants presenting with hallux rigidus was 3 (range 1 – 3). The overall proportion of participants with bilateral pathology was 53% (64 out of 120), however there was a difference between males and females. Of the females recruited into the study, 57% (56 out of 99) presented with bilateral pathology, compared to only 38% (8 out of 21) of males.

**Figure 1:**
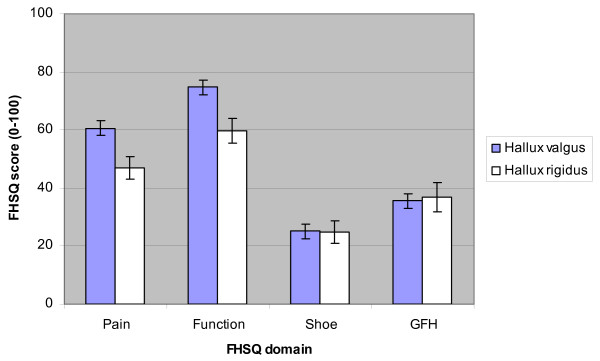
Comparison between mean hallux valgus and hallux rigidus Foot Health Status Questionnaire (FHSQ) domain scores (error bars represent standard error).

With respect to the outcomes measured on the Foot Health Status Questionnaire, there were two significant findings. Firstly, there was a statistically significant difference between hallux rigidus and hallux valgus (p < 0.01) for pain. Secondly, there was a statistically significant difference between hallux rigidus and hallux valgus (p < 0.01) for function. A comparison of the Foot Health Status Questionnaire scores (four foot domains) for hallux valgus versus hallux rigidus is provided in Table 3. To highlight differences between hallux valgus and hallux rigidus we have graphically presented these findings in Figure 1.

**Table 3:** A comparison between hallux valgus and hallux limitus, including the mean differences, for each of the four domains of the Foot Health Status Questionnaire (0–100 scale)

	**Hallux Valgus – mean ± SD (n = 82)**	**Hallux Rigidus – mean ± SD (n = 38)**	**Mean difference Between groups**	**95% CI**	**p value**
Pain	60.6 (± 22.9)	46.9 (± 23.6)	13.8	4.6 to 22.9	<0.01
Function	74.7 (± 22.9)	59.7 (± 25.5)	15.0	5.3 to 24.7	<0.01
Shoe fit	25.1 (± 23.1)	24.8 (± 23.8)	0.3	8.9 to 9.6	0.95
GFH	35.5 (± 23.0)	36.7 (± 31.0)	1.1	10.1 to 12.4	0.84

Note: CI = confidence interval, GFH = General Foot Health

## Discussion

The aim of this study was to compare the effects of hallux valgus and hallux rigidus on foot health status (health-related quality of life) in participants seeking surgical opinion. Such a benchmarking activity has not previously been reported. Comparing the impact of hallux valgus and hallux rigidus on foot health status is important, as accurate diagnosis and classification is not straight forward. The pain and deformity of hallux rigidus can clinically mimic hallux valgus, and the deformity of hallux valgus can result in arthrosis, similar to hallux rigidus. The difficulty in diagnosis and classification can be illustrated using the following examples from the literature. Coughlin and colleagues acknowledged that some patients having undergone surgery for hallux valgus were later excluded from their study of hallux valgus due to the presence of arthritis [20]. While in another study, 12% of included patients in a study of hallux rigidus [14] presented with radiological findings indicative of hallux valgus.

### Impact of hallux valgus and hallux rigidus on health-related quality of life

Previous studies have shown that hallux valgus has a significant and detrimental effect on various parameters of *generic *health-related quality of life [2,12], however no studies have been identified that compare hallux valgus and hallux rigidus, particularly in relation to their effect on *foot-specific *health-related quality of life (e.g. using the Foot Health Status Questionnaire).

The Foot Health Status Questionnaire was designed as a measurement instrument for outcomes studies, where an intervention is given and the outcome of interest is measured before and after administration of the intervention. However, it can also be used to compare health-related quality of life across conditions affecting the feet (e.g. hallux valgus to hallux rigidus). Ideal values in each domain are represented by the score 100 (0 – 100 scale). The authors are unaware of any normative data for individuals who have no pathology in the great toe joint. In this study, the Foot Health Status Questionnaire scores for hallux valgus were well below the ideal of 100 (60.6 for pain, 74.7 for function, 25.1 for shoe fit and 35.5 for general foot health). Similarly, for hallux rigidus the mean scores were also well below the ideal (46.9 for pain, 59.7 for function, 24.8 for shoes and 36.7 for general foot health).

While hallux valgus and hallux rigidus both cause significant reduction in health-related quality of life, our results clearly demonstrate that hallux rigidus is a more debilitating condition than hallux valgus in a cohort seeking surgical advice from a podiatric surgeon. Importantly, there was no significant difference in age – which would be associated with the Foot Health Status Questionnaire results – between the hallux valgus and hallux rigidus groups. This rules out this potential confounder (i.e. that one group might have had worse health-related quality of life simply because they were on average older) and strengthens our findings. The findings from this study, therefore, provide evidence that hallux rigidus has a greater negative impact compared to hallux valgus in the key areas of pain and function. The lack of significant difference between the two conditions in respect of footwear and general foot health reflects that although there are distinct differences in pain and function, there are also some similarities. Alternatively, it is possible that the shoe fit and general foot health domains of the Foot Health Status Questionnaire are not sufficiently sensitive to detect clinically worthwhile differences between the two conditions. The authors are not aware of any previous studies which have reported the relative impacts of these conditions on these domains. These findings are of interest and further research would be of use to explore both the apparent similarities in impact on foot health of these conditions and the sensitivity of the footwear and general foot health domains of the Foot Health Status Questionnaire for detecting differences between hallux valgus and hallux rigidus.

These findings lend support to a growing body of research suggesting that great toe joint pathology is a significant health issue [6,7,12]. Our findings add to this body of research by demonstrating that some forms of great toe joint pathology have greater impact than others.

### Prevalence and gender distribution of hallux valgus and rigidus

It is commonly reported that hallux valgus has a high prevalence within the community and particularly amongst women [1]. Little has been reported on the prevalence of hallux rigidus. In this study hallux valgus was the more common presenting pathology (68% hallux valgus versus 32% hallux rigidus). With respect to gender distribution, women were disproportionately represented (82%) in the overall cohort. These findings are generally consistent with the literature [5-9,11,21].

Of further interest is the proportion of participants with hallux valgus and hallux rigidus within males and females, indicating a possible gender bias of each condition in patients presenting to podiatric surgeons. In the male cohort, 33% presented with hallux valgus, whereas 76% of the female cohort presented with the same condition. In contrast, 67% of the males presented with hallux rigidus, whereas only 24% of the females presented with this pathology. Although one study [14] reports females as more commonly presenting with hallux rigidus, the proportion of males presenting with hallux rigidus in our study is supported by earlier reports of 60% to 64% [21-23]. Variations in gender predisposition reported in the surgical literature may not necessarily indicate that females develop these conditions more commonly, it may simply show that females are more likely to present for surgical advice than men.

### Limitations of the study

There are a number of limitations that need to be considered with respect to this study. Firstly, there was no control group of participants with absent great toe joint pathology. A case-control study would provide a more accurate representation of impact of the conditions relative to normative population values. Secondly, diagnosis of participants in this study was dependent on a clinical diagnosis, including x-rays, made by the participating surgeons. As there were seven surgeons involved in collecting data, some individual variation may have occurred in the accuracy of the data collected that related to the diagnosis and the coding of the diagnosis. To reduce this potential for error, only experienced foot surgeons were recruited and training was provided to each surgeon on the classification systems used in the study. Thirdly, the findings from this study reflect the status of patients in Australia presenting to foot surgeons and caution is needed generalising these findings to the wider population. Finally, some criticisms have been raised about the Foot Health Status Questionnaire, including its initial development, validation and its ability to discriminate levels of general foot health [24,25]. However, in a comparison with other foot and ankle outcomes by Suk and colleagues [26] the Foot Health Status Questionnaire was rated the highest in quality (methodological quality and clinical utility) of 25 foot and ankle outcome measures.

## Conclusion

This study has detected measurable differences in foot health status between hallux valgus and hallux rigidus in patients presenting to foot surgeons in Australia. While both appear to have a negative impact on health status, our findings suggest hallux rigidus has a more significant impact on pain and function. The Foot Health Status Questionnaire was shown to be a sensitive measure that is able to distinguish these differences. Finally, this study found a greater prevalence of hallux valgus in women, but a greater prevalence of hallux rigidus in men.

## Competing interests

KBL is a Deputy Editor of the Journal of Foot and Ankle Research. It is journal policy that editors are removed from the peer review and editorial decision making processes for papers they have co-authored.

## Authors' contributions

MG designed the study, supervised data collection, performed the data analysis and wrote the manuscript. KL assisted with the data analysis and writing of the manuscript. PR assisted with writing of the manuscript.
